# Acute-Onset Retinal Conditions Mimicking Acute Optic Neuritis: Overview and Differential Diagnosis

**DOI:** 10.3390/jcm12175720

**Published:** 2023-09-01

**Authors:** Emanuela Interlandi, Francesco Pellegrini, Chiara Giuffrè, Daniele Cirone, Daniele Brocca, Andrew G. Lee, Giuseppe Casalino

**Affiliations:** 1 Department of Ophthalmology, “Ospedale del Mare”, ASL Napoli 1-Centro, 80147 Naples, Italy; 2 Department of Ophthalmology, “Santo Spirito” Hospital, 65122 Pescara, Italy; francepellegrini@virgilio.it; 3 Centro Europeo di Oftalmologia, 90141 Palermo, Italy; giuffrechiara10@gmail.com; 4 Ophthalmology Department, San Raffaele Scientific Institute, University Vita-Salute, 20132 Milan, Italy; 5 Department of Ophthalmology, “Villa Anna” Hospital, 63074 San Benedetto del Tronto, Italy; ciro.dan@inwind.it; 6 Department of Ophthalmology, “De Gironcoli” Hospital, AULSS2 Marca Trevigiana, 31015 Conegliano, Italy; daniele.brocca@aulss2.veneto.it; 7 Department of Ophthalmology, Blanton Eye Institute, Houston Methodist Hospital, Houston, TX 77030, USA; aglee@houstonmethodist.org; 8 Departments of Ophthalmology, Neurology and Neurosurgery, Weill Cornell Medicine, New York, NY 10021, USA; 9 Department of Ophthalmology, University of Texas Medical Branch, Galveston, TX 77555, USA; 10 University of Texas MD Anderson Cancer Center, Houston, TX 77030, USA; 11 Texas A and M College of Medicine, Bryan, TX 77807, USA; 12 Department of Ophthalmology, The University of Iowa Hospitals and Clinics, Iowa City, IA 52242, USA; 13 Fondazione IRCCS Cà Granda, Ospedale Maggiore Policlinico, University of Milan, 20122 Milan, Italy; peppecasalino@gmail.com

**Keywords:** acute-onset visual loss, optic neuritis, retinal conditions, multimodal imaging, differential diagnosis

## Abstract

Acute optic neuritis (AON) is a common cause of sudden visual loss in young patients. Because of the risk of demyelinating disease, patients affected by unilateral or bilateral optic neuritis should be evaluated and treated accordingly. Despite advancements in imaging of the brain and retina, misdiagnosis of AON is not uncommon. Indeed, some acute disorders of the retina have the potential to mimic AON and their prompt diagnosis may avoid unnecessary neurologic investigation, psychological stress to the patient, and delays in treatment. This review describes uncommon retinal disorders presenting with sudden-onset visual loss and absent or subtle funduscopic manifestation that can mimic AON. Multimodal retinal imaging is essential in detecting these conditions and in their differential diagnosis. It behooves neurologists and general ophthalmologists to be aware of these entities and be familiar with multimodal imaging of the retina.

## 1. Introduction

Sudden-onset visual loss may result from a number of lesions along the anterior visual pathway and traditionally a comprehensive ophthalmic evaluation including fundus examination was usually sufficient to detect the site of the lesion. When the ophthalmic examination is unrevealing a patient may be referred to a neurologist or a neuro-ophthalmologist with the suspicion of acute optic neuritis (AON) or other neurological disorders, not uncommonly after a number of costly procedures are performed, such as brain magnetic resonance imaging (MRI). However, some acute retinal diseases may present with symptoms (e.g., visual acuity or visual field loss) and signs (e.g., relative afferent pupillary defect and a normal or swollen optic nerve) that can mimic AON. These retinal conditions may present with subtle or no apparent changes on fundus examination [1].

Recent advances in non-invasive multimodal retinal imaging, especially high-resolution optical coherence tomography (OCT) and fundus autofluorescence (FAF) make it possible to identify the typical features of these retinal conditions [2].

Neurologists and general ophthalmologists should be aware of those unilateral or bilateral retinal conditions able to mimic AON and always consider multimodal retinal imaging in the differential diagnosis process [3].

This narrative review will focus on those retinal conditions with an acute onset that may be misdiagnosed as AON and will discuss their presentation on retinal imaging and their differential diagnosis. These disorders are presumed to be either inflammatory or ischemic in nature and have a so far poorly understood pathophysiology.

## 2. Results

We have identified a group of different acquired retinal disorders with a sudden onset of symptoms that are mostly affecting young adults. History, clinical characteristics, and fundus findings are paramount but not always sufficient to detect these retinal disorders. We first detail the symptoms and signs that may suggest a retinal disorder and we then detail each of these retinal conditions focusing on their presentation and multimodal retinal imaging features.

- Retinal phosphenes: more visible in the dark, are commonly caused by vitreous traction on the retina. However retinal inflammation may also produce flickering and shimmering. Phosphenes may rarely occur in optic neuritis [2] but pain upon eye movement is usually present. They are common in migraines (visual auras) but are homonymous and unaffected by light levels. They may respect a typical pattern (teichopsia) often easily recognized by patients themselves.
- Metamorphopsia: distortion of images is a clear indicator of a maculopathy as the cause of visual loss. Straight lines appear wavy and may be accompanied by central or paracentral scotoma and micropsia (objects look smaller if compared to an unaffected eye).
- Blind spot enlargement: if a blind spot enlargement is seen on the visual field test, a retinal disorder affecting the peripapillary retina should be suspected, especially in the absence of optic disc swelling. Optic disc edema usually does not cause such enlargement unless it is very severe.
- Photo stress test: after exposure to bright light, there is a slow recovery in cases of retinal disorder affecting the cone function.
- Absence of relative afferent pupillary defect (RAPD): optic neuropathy (such as optic neuritis) is associated with RAPD unless bilateral and symmetric. Thus, visual loss in the absence of RAPD usually points to the macula as the site of the lesion. However, one must remember that disorders of the peripapillary retina may be accompanied by mild disc swelling and a RAPD [3].
- Lack of pain with eye movement: the typical presentation for AON is a young female with acute loss of vision, evidence for an optic neuropathy (e.g., loss of visual acuity and visual field defect associated with a RAPD), pain with eye movement, and a normal fundus. The absence of pain with eye movement should raise suspicion for an alternative diagnosis (atypical optic neuritis, acute-onset retinopathy, ischemic/inflammatory/compressive optic neuropathy, among others).

### 2.1. Acute Idiopathic Maculopathy

Acute idiopathic maculopathy (AIM) is a rare and rapidly evolving macular condition first described by Yannuzzi et al. in 1991 [4]. Originally considered unilateral, further features such as papillitis, eccentric lesions, and bilaterality were reported in 1996 as part of the extended spectrum of the disease [5]. AIM commonly affects young healthy adults with a prodromal flu-like illness. No prevalence of sex is reported.

Despite how the etiology remains unclear, several reports suggest that coxsackievirus infection or vaccination may play a certain role [6,7,8,9]. AIM typically presents with severe visual loss (20/200 or even worse) and central scotoma. Fundus examination may reveal irregular macular neurosensory detachment overlying a greyish or yellowish thickening of the retinal pigment epithelium (RPE). Occasionally retinal hemorrhages and inflammatory cells in the posterior vitreous may be part of the posterior segment findings.

A disease staging based on OCT and different FAF patterns has been recently proposed by Pajtler Rosar et al. [10]. Pattern 1 (central hypo-autofluorescence with surrounding hyper-autofluorescence on the border with the uninvolved retina) and Pattern 2 (predominant stippled hyper-autofluorescence and coexisting hypo-autofluorescence) were found at presentation or during the first weeks since the onset of symptoms. Pattern 3 (central hyper-autofluorescence surrounded by hypo-autofluorescence) and Pattern 4 (homogeneously decreased autofluorescence) were observed during the disease course or in the recovery phase. Patterns 1 and 2 were associated with decreased visual acuity and outer retinal disruption on OCT. Patterns 3 and 4 were associated with a restored visual acuity and good integrity of the outer retinal layers on OCT. In the recovery phase, RPE changes are observed as the central hyperpigmentation subsides and a final hypopigmentation with a “bull’s-eye” appearance of the macula is observed.

**Figure 1:**
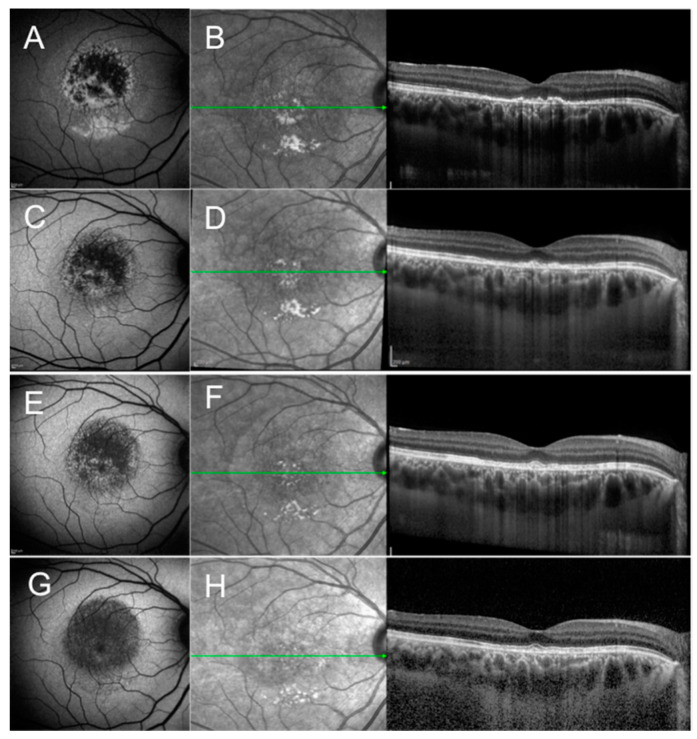
Fundus autofluorescence (FAF) and optical coherence tomography (OCT) scan of a case of acute idiopathic maculopathy of the right eye at presentation (**A**,**B**), 4 weeks after presentation (**C**,**D**), 8 weeks after presentation (**E**,**F**), and 12 weeks (**G**,**H**) after presentation. (**A**) FAF shows a predominant hypoautofluorescent signal surrounded by a hyperautofluorescent signal (Pattern 1 of the proposed disease Stage 1); (**B**) OCT shows disruption of the retinal pigment epithelium (RPE) and outer retinal layers with increased choroidal transmission; (**C**–**H**) Subsequent FAF and OCT scan during follow-up show decreasing intensity of the hypoautofluorescent signal and gradual improvement of the integrity of RPE and outer retinal layers, respectively. (**G**) Two years after presentation, FAF shows homogeneously decreased FAF (Pattern 4 of the proposed disease Stage 4).

The disease is self-limiting and on average it takes 4 months (range 1–7 months) for the vision to recover and recurrences are rare [10,11]. OCT in the early phase may show a detached macula, with possible bacillary layer detachment, disruption of the outer retinal layers, and usually an intact external limiting membrane (ELM) (Figure 1). Later in the disease course, outer retinal layers mostly recover accompanied by vision improvement. Focal areas of either RPE thickening and/or RPE attenuation develop, consistent with choroidal hypo- and/or hyper-transmission on OCT, respectively.

Jung et al. [12] postulated that the ELM integrity is crucial to the outer photoreceptor layers healing process and thus for vision improvement. Near-infrared (NIR) reflectance imaging shows an irregular and granular pattern of the affected macula, which persists even if vision has improved. Fluorescein angiography (FA) shows irregular hyper-fluorescence in the early angiograms and a two-level hyper-fluorescence in the late phases from the staining of the thickened RPE and pooling of the dye within the detached neuro-epithelium. Several groups investigated the role of the choroid in the pathogenesis of AIM with indocyanine green angiography (ICGA) and enhanced depth imaging OCT (EDI-OCT). Early ICGA findings are represented by a hypo-fluorescent area, eventually larger than the clinically visible lesion, dilated choroidal vessels, and neighboring moth-eaten vessels [12,13].

Srour et al. [14] described the very early stage (first three hours) of the disease with EDI-OCT showing increased subfoveal choroidal thickness. In 2015, Hashimoto and colleagues [15] in their laser speckle flowgraphy study demonstrated impaired choroidal blood flow velocity in the early phase with subsequent improvement during the recovery phase. Similar findings have been reported by Fernandez-Avellaneda through OCT-angiography analysis of the choriocapillaris [16]. The authors hypothesized a combination of inflammation and ischemia of the choroid may lead to RPE decompensation and subretinal fluid exudation.

AIM must be differentiated from other macular conditions. Central serous chorioretinopathy mostly affects healthy middle-aged adults, usually with anxiety, and shows neurosensory detachment of the macula in the acute phase. Unlike AIM, prodromal illness is absent and recurrences are common. FAF may show multifocal areas of decreased and increased signal and FA shows one or more RPE leaks with choroid hyper-permeability on ICGA. Acute posterior multifocal placoid pigment epitheliopathy (APMPPE) is characterized by multifocal placoid lesions at the posterior pole and mid periphery, typically affecting both eyes, most commonly with foveal sparing. While in the early phases FA and ICGA show both hypofluorescence, in the late phases FA shows hyperfluorescence while ICGA keeps showing hypofluorescence (FA-ICGA dissociation) [17]. APMPPE is a self-limiting condition but typically leaves behind multifocal RPE scarring. Since choroidal neovascularization (CNV) may be a possible complication of AIM [4,18], extensive multimodal retinal imaging including FA, ICGA, and possibly OCTA is advisable in cases of presumed AIM.

### 2.2. Acute Retinal Pigment Epitheliitis

Acute retinal pigment epitheliitis (ARPE), also named Krill’s disease, was first described by Krill and Deutman in 1972 [19]. It is a rare, idiopathic, and self-limiting inflammatory disease of the retina. Because of its rarity, the demographic and clinical findings of the patients with ARPE are not fully understood but it seems to affect young healthy individuals with no racial predisposition and a slight female predominance [20,21].

Etiology and pathophysiology remain unknown, but flu-like symptoms may precede the onset of ARPE, suggesting a correlation between viral infections and the disease [20]. Patients generally complain of painless blurred vision, central scotoma, and metamorphopsia with acute onset in one eye, although bilateral involvement can occasionally occur [21]. Visual acuity generally is around 20/40 and may range from 20/30 to 20/100 [19]. The condition presents rapid resolution with full or almost complete recovery within a few weeks without treatment. Recurrences are also possible [22,23,24]. The characteristic funduscopic appearance of ARPE is a fine pigment stippling at the center of the macula surrounded by a yellow whitish hypopigmented halo.

**Figure 2:**
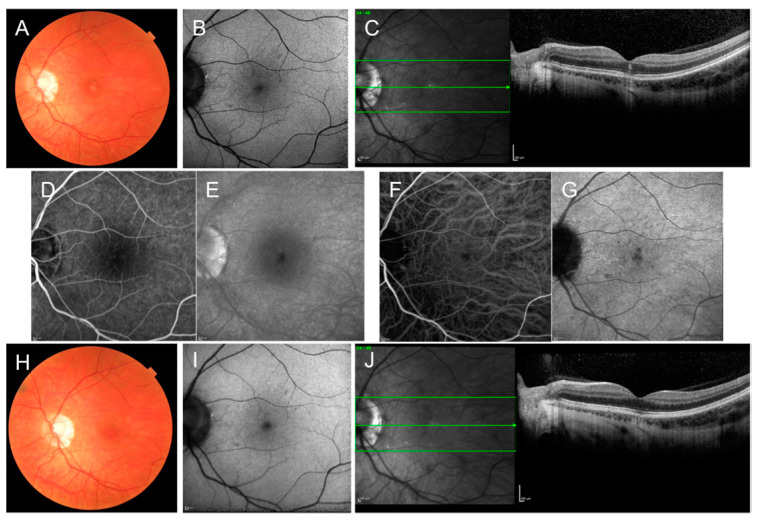
Multimodal retinal imaging of a case of acute retinal pigment epitheliitis of the left eye at presentation (**A**–**G**) and after 6 weeks (**H**–**J**). (**A**) Color fundus photograph at presentation shows abnormal foveal reflex; (**B**) Fundus autofluorescence (AF) shows absence of AF abnormalities; (**C**) Optical coherence tomography (OCT) scan shows subfoveal retinal pigment epithelium and outer retinal disruption; (**D**–**G**) Fluorescein angiography (**D**,**E**) and indocyanine green angiography (**F**,**G**) in the early and late frames do not show obvious abnormalities of the dyes circulation. (**H**) Color fundus photograph six weeks after presentation shows improvement of the foveal reflex; (**I**) AF findings were stable; (**J**) OCT scan shows recovery of the reflectivity of the subfoveal RPE and outer retinal layers.

Previously thought to be an inflammatory disease of the RPE, recent studies using high-resolution OCT have revealed that the primary inflammation area in ARPE is located at the outer retinal layers, with abnormal hyperreflectivity, especially involving the interdigitation zone (IZ), ellipsoid zone (EZ), and ELM (Figure 2) [20,21]. FAF may show mild hyperautofluorescence in the involved area [21,25]. FA usually reveals transmission hyperfluorescent defects without leakage [21]. ICGA may show a hypocyanescent lesion surrounded by a hypercyanescent halo with a cockade-like pattern or a patchy hypercyanescence [21]. Central amplitudes in multifocal electroretinogram (mfERG) are depressed in patients with ARPE [26]. On the visual field test, the threshold sensitivity is decreased in the regions corresponding to the macular lesions [27].

Differential diagnosis includes the most frequent causes of acute, unilateral visual loss in young adults, including AON, multiple evanescent white dot syndrome (MEWDS), acute macular neuroretinopathy (AMN), and AIM. AON is the most common cause of acute unilateral visual loss in young people, with female predilection. Unlike ARPE, in AON the acute visual loss is accompanied by pain and the examination of patients typically reveals visual field loss, color vision defect, and a RAPD in the involved eye. The funduscopic examination in AON is generally normal, with less than 25% of patients presenting with optic disc swelling [28]. The macular lesions observed in ARPE are absent in AON.

MEWDS is a rare primary inflammatory choriocapillaropathy, part of the so-called “white dot syndromes”, affecting young women most frequently. Like ARPE, MEWDS is a self-limiting condition characterized by prodromal influence-like episodes and unilateral decreased vision or scotoma. In contrast to ARPE, MEWDS is not confined to the macula and its typical fundus findings include foveal granularity and white dot lesions extending from the posterior pole and the peripapillary area to the equator. FA and ICGA are useful in the differential diagnosis with ARPE. In MEWDS, FA shows diffuse punctate hyperfluorescence, whereas ICGA reveals multiple hypocyanescent dots that can outnumber the lesions seen clinically or with FA [29].

AMN is characterized by reddish-brown paracentral lesions in a petaloid or tear-drop configuration pointing toward the fovea. Similar to ARPE, patients experience sudden central or paracentral scotoma. However, OCT in AMN shows the disruption of EZ and hyperreflective plaques at the border of the outer plexiform layer and outer nuclear layer, possibly due to ischemia of the deep retinal capillary plexus [30].

ARPE requires no treatment because of its self-limiting nature. The prognosis is excellent and a complete functional recovery with disappearance of the fundus and OCT abnormalities is expected within a few weeks. However, incomplete visual recovery and persistent disruption of the EZ have been reported [19,31].

### 2.3. Acute Macular Neuroretinopathy

First described by Bos and Deutman in 1975, acute macular neuroretinopathy (AMN) is a rare disease characterized by darkish brown-red, wedge-shaped dots in the macula pointing to the fovea [32]. Since the lesions do not typically involve the fovea, visual acuity is generally unaffected and most commonly patients complain of paracentral scotomas; blurred vision, floaters, and metamorphopsia have also been reported [30]. AMN affects most frequently young and Caucasian women and is bilateral in 54.4% of cases. Many associations or risk factors have been identified, including fever, use of oral contraceptives, exposure to either epinephrine or ephedrine, trauma, systemic shock, intravenous contrast exposure, pre-eclampsia, post-partum hypotension, use of cocaine, and caffeine consumption [30]. Several cases following COVID-19 vaccination have also been reported [33].

The pathogenesis of AMN is unclear, but recent studies using OCT point to deep capillary plexus (DCP) ischemia as a possible pathogenic mechanism [34]. One single case of AMN in association with MEWDS has been described [35]. Of note, AMN has been reported in a patient with acute myeloid leukemia (AML) and deceased by COVID-19. This case supports the role of ischemia in the outer retina, of which AML may contribute to the pathological mechanism [36]. Since the incidence of AMN increased during the COVID-19 pandemic years, a causative link is possible and different pathogenetic hypotheses common to COVID-19 respiratory disease pathogenic mechanisms, such as coagulation anomalies, hyperinflammation, and immune dysregulation, have been postulated [37].

**Figure 3:**
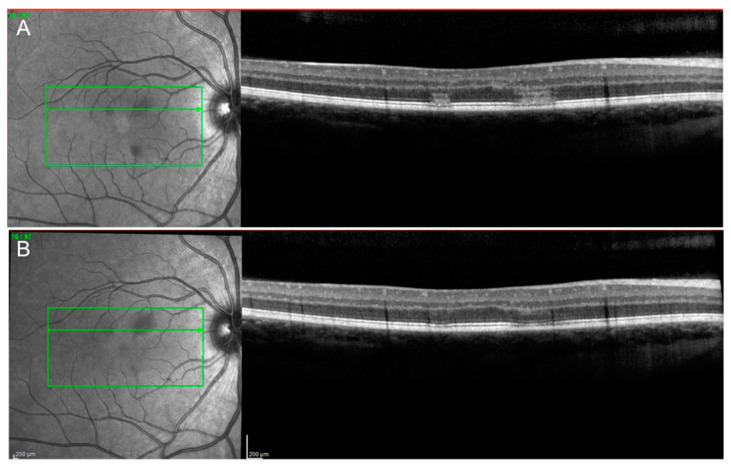
Near-infrared reflectance imaging (NIR) and simultaneous optical coherence tomography (OCT) scan of a case of acute macular neuroretinopathy (AMN) of the right eye at presentation (**A**) and 4 weeks after presentation (**B**). (**A**) OCT scan passing through the tear-shaped AMN lesions visible on NIR reveals increased reflectivity of the outer plexiform layer and outer nuclear layer with disruption of the ellipsoid zone. (**B**) Follow-up OCT scan shows attenuation of the ellipsoid zone and overlying thinning of the outer nuclear layer.

**Figure 4:**
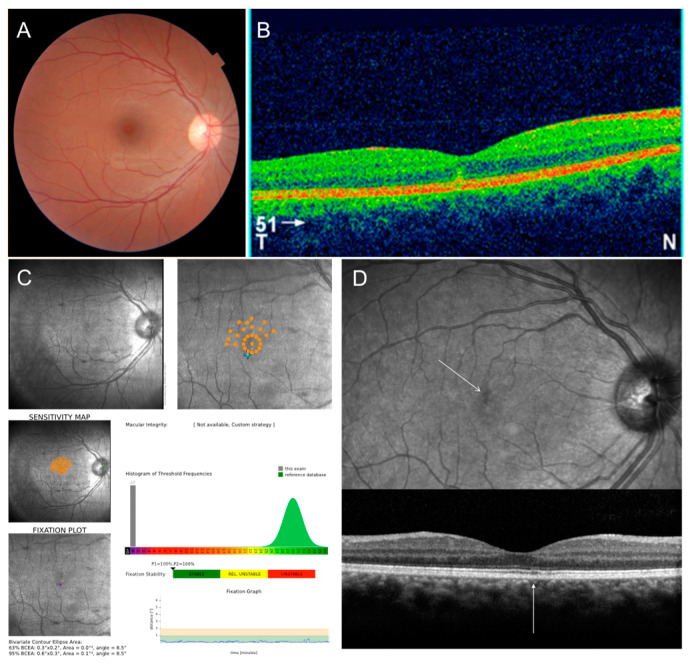
Acute macular neuroretinopathy (AMN) of the right eye at presentation (**A**,**B**) and 2 weeks after presentation (**C**,**D**). (**A**) Color fundus photography is unremarkable, (**B**) OCT scan shows a small juxtafoveal hyperreflectivity in the outer retina. (**C**) Microperimetry reveals a focal depression of the macular sensitivity; (**D**) OCT scan shows attenuation of the ellipsoid zone in correspondence of a tear-shaped hyporeflective area visible in NIR (white arrow).

The typical wedge-shaped lesions are arranged in a petaloid, oval, or tear-drop configuration. In some cases, they are not clinically identifiable and multimodal imaging is necessary to reveal paracentral macular changes (Figure 3 and Figure 4). Rarely, superficial retinal hemorrhages and macular edema can be associated.

OCT and NIR reflectance imaging represent the most sensitive imaging techniques to diagnose and follow AMN patients, even when clinical examination is normal. The most common finding seen on OCT is EZ disruption; hyperreflective plaques at the border of the outer nuclear layer (ONL) and the outer plexiform layer (OPL), IZ disruption, and RPE abnormalities may also be detectable. With time, EZ generally reconstitutes but IZ disruption may persist. The hyporeflective changes shown by NIR correlate with EZ and IZ disruption on OCT and the latter could represent a subclinical derangement of RPE melanin [38]. Normalization of IZ on OCT and the fading of dark NIR lesions are associated. OCT angiography (OCTA) confirmed inner choroidal vascular flow void as a possible pathogenetic process of AMN and focal impairment of the DCP within the distinctive lesions [39]. Using FAF, AMN changes have been described either as subtle areas of mild hypoautofluorescence or focal hyperautofluorescence correlated with EZ loss observed on OCT [40,41]. FA is generally normal, however, hypofluorescence of the lesions is the most frequent abnormality detectable. Similarly, ICGA images are usually normal but early hypocyanescence can be noted.

Only a small percentage of patients with AMN that undergo full-field electroretinography (ffERG) show abnormalities, including reduced a-wave and/or b-wave and diminished rod and cone response. mfERG is a more sensitive electrophysiology testing for patients with AMN. It usually reveals most frequently reduced amplitudes and occasionally diminished implicit time. Visual field testing shows paracentral scotomas consistent with AMN lesions.

AMN should be differentiated from other entities, such as ARPE, AON, and white dot syndromes, including MEWDS and APMPPE. Interestingly, a new association between AMN and AON has been recently reported [42]. The occurrence of AMN in AON could be explained by inflammatory edema compressing retinal arterial vessels and reducing blood flow, particularly in the DCP. The patients complain of large scotomas and a multimodal imaging approach is essential to detect typical AMN lesions in patients with AON. Hence, it is likely that this association has been underestimated in the past. AMN has a variable prognosis [43].

Although AMN has a self-limiting course and outer retinal recovery is observed, usually patients complain of persistent scotomas with good visual acuity. To date, there is no causative treatment for AMN and only an observational approach is recommended.

### 2.4. Paracentral Acute Middle Maculopathy

**Figure 5:**
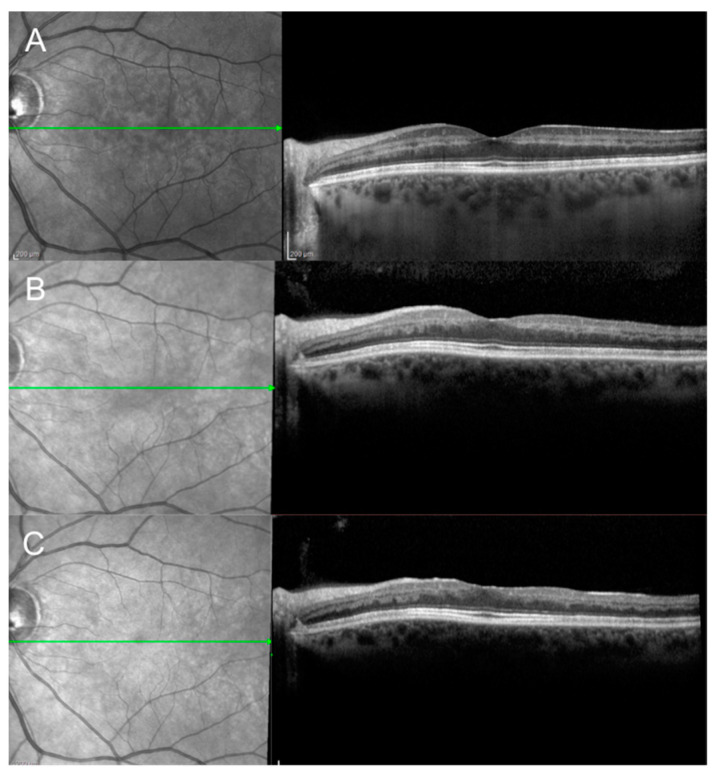
Near-infrared reflectance (NIR) imaging and simultaneous optical coherence tomography (OCT) scan of a case of paracentral acute middle maculopathy (PAMM) of the left eye at presentation (**A**) 2 weeks after presentation (**B**) and 8 weeks after presentation (**C**). (**A**) OCT scan shows hyperreflective band-like lesions involving the middle layers of the retina at the level of the inner nuclear layer (INL). (**B**,**C**) Follow-up OCT scan shows decreased reflectivity and subsequent thinning of the INL.

Paracentral acute middle maculopathy (PAMM) is an OCT manifestation presenting with hyper-reflective band-like lesions involving the outer plexiform layer/inner nuclear layer (OPL/INL) junction resulting in permanent INL thinning and variable vision loss (Figure 5) [44].

PAMM has been considered a possible variant of AMN first reported by Sarraf et al. in 2013 [44]. Using OCT, the authors described two types of disease: AMN Type 1 (or PAMM) and AMN Type 2 (classical AMN). With AMN, PAMM seems to have a common pathophysiology of retinal microvascular ischemia, most likely at the level of the intermediate capillary plexus (ICP) and DCP. However, in PAMM the lesions are more superficial than AMN, at the level of the INL. Nowadays, it has been established that PAMM and AMN are two distinct entities with similar pathophysiology and some overlapping findings [45].

Patients with PAMM present with sudden onset of one or more paracentral scotomas in one or both eyes. Visual acuity can be normal, mildly, or severely decreased. Fundus examination may show either no significant abnormalities or subtle parafoveal lesions that usually appear deeper, smoother, and greyer than common cotton wool spots. The main age of presentation is 49–53 years with no gender predilection [46].

PAMM can be either associated with several conditions, such as retinal vascular diseases and other ophthalmic conditions, systemic diseases, vasculopathic and environmental risk factors, or idiopathic. In the latter case, PAMM usually affects younger, female, and otherwise healthy individuals. A recent history of flu-like episodes, post-H1N1 vaccination status, and pregnancy have been suggested as possible contributing factors to the development of the disease [44,47,48].

Many retinal vascular diseases have been related to PAMM, including arterial or venous occlusion, Purtscher’s retinopathy, sickle cell retinopathy (SCR), inflammatory occlusive vasculitis, hypertensive retinopathy, and diabetic retinopathy [46,47]. These associations provide further support for the notion that vascular dysfunction may play an important role in PAMM.

PAMM has also been associated with other ophthalmic conditions, such as inflammatory chorioretinopathies, congenital glaucoma, foveal hypoplasia (FH), juvenile dermatomyositis, ophthalmic surgeries, and eye compression injury [47]. Systemic conditions linked to PAMM include neurologic diseases, carotid arterial disease, vascular surgeries, Susac syndrome, giant cell arteritis, and others [46].

Iovino et al. analyzed coincident or sequential PAMM and AMN lesions in the same eye in patients with retinal vein occlusion, Purtscher’s retinopathy, and retinal phlebitis suggesting a common pathogenic pathway that may result from impairment of the venous outflow and hypoperfusion at the level of the DCP, possibly leading to Müller cell injury in the Henle’s fiber layer [49].

A few cases of PAMM and/or AMN have been reported among patients with COVID-19 and after COVID-19 vaccination [50].

Recently, a novel variant of PAMM, called central acute middle maculopathy (CAMM), has been recognized in foveal hypoplasia [51]. In CAMM, the lesion also progresses from a hyperreflective band to thinning at the level of the INL. In this atrophic stage, a ‘pseudo-foveal pit’ is visible, often in addition to mild disruption of EZ and IZ.

In patients with PAMM, swept-source (SS)-OCT may reveal undulations of the margins of the characteristic band-like hyperreflectivity in the INL, as a sign of reperfusion and resolution of microvascular infarction [52]. With OCTA, it is possible to assess the extent of retinal ischemia in the ICP and DCP. Simultaneous en face OCT and OCTA are emerging as the preferred method to image and follow patients with PAMM [53].

With en face OCTA, three patterns of distribution of PAMM associated with retinal microvascular ischemia have been identified: 1. sectoral distribution, coherent with transient occlusion of a large arteriolar vessel with rapid restoration of blood flow; 2. globular pattern, as one or multiple ovoid hyperreflective patches probably due to precapillary occlusion related to SCR or embolic events; 3. fern-like pattern of hyperreflectivity that may be seen in central retinal vein occlusion, as a result of stasis and hypo-oxygenation of total retinal circulation due to perivenular ischemia [53]. En face swept-source OCTA has been shown to have a superior depth and field of view than en face spectral-domain OCTA [54]. En face SS-OCTA allows for us to measure the extent of retinal ischemia in PAMM, which is related to INL atrophy, and for the observation of the dynamic course of the disease. NIR is a useful tool for diagnosis of PAMM, revealing dark paracentral lesions. FAF shows corresponding hypoautofuorescent lesions. In the CAMM variant patients, foveal hyporreflectivity on NIR and globular-shaped hypoautofluorescent lesions have been observed [51]. FA cannot easily distinguish the ICP from DCP, but it may denote findings correlated to the underlying etiology.

An appropriate diagnostic work-up should be individualized to rule out cardiovascular risk factors, taking into account the patient’s past medical history and their systemic and ocular findings. As first level examination, it is advisable to prescribe a complete blood cell count, metabolic and lipid panel, screening for hypercoagulability, infectious agents, autoimmune and inflammatory markers, blood pressure, echocardiography, and carotid Doppler ultrasonography.

The main differential diagnosis of PAMM remains AMN; as discussed above, the characteristic fundus appearance and the deeper lesions at the junction of the OPL and ONL detectable by OCT may help to easily differentiate AMN from PAMM.

The prognosis of PAMM is variable, and its natural course is not clearly understood. Several patients may have INL thinning, ONL thickening, abnormal vasculature (especially in the DCP), and, rarely, focal serous retinal detachment with persistent scotomas and affected visual acuity, but spontaneous improvement has been reported [55]. No treatment is recommended for PAMM. Nevertheless, treatment of the underlying cause, when present, must be considered.

### 2.5. Acute Zonal Occult Outer Retinopathy—Acute Annular Outer Retinopathy Variant

**Figure 6:**
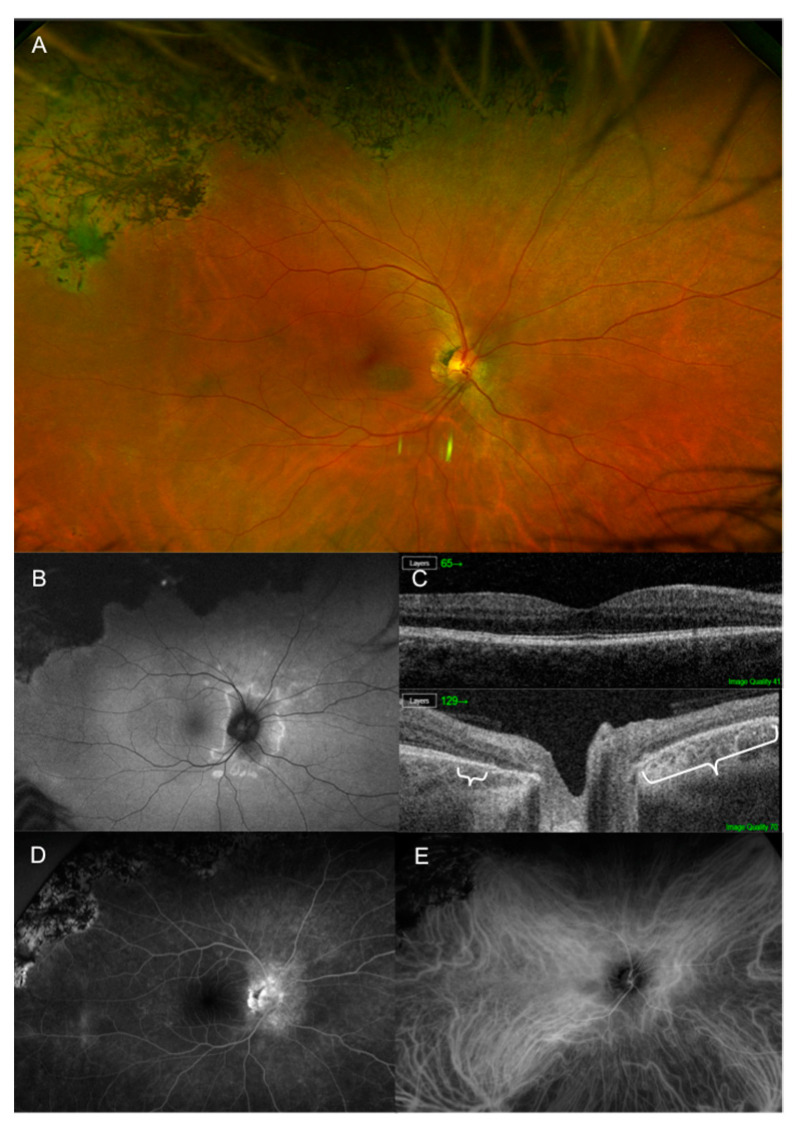
Multimodal imaging of acute zonal occult outer retinopathy, variant acute annular outer retinopathy of the right eye. (**A**) Pseudocolor fundus photography shows peripapillary atrophy and pigmentary changes in the superior periphery. (**B**) Ultra-widefield autofluorescence shows a hyperautofluorescent ring around the optic nerve and hypofluorescent lesions in the superior periphery. (**C**) Structural optical coherence tomography scan centered on the fovea (top image) and on the optic nerve (bottom image) shows the loss of the peripapillary photoreceptor layers (white curly brackets). (**D**) Fluorescein angiography shows a hyperfluorescent annular lesion around the optic nerve and hypo/hyperfluorescent lesions in the superior periphery. (**E**) Indocyanine green angiography shows mild hypofluorescence around the optic nerve and hypofluorescent lesions in the superior periphery.

Acute annular outer retinopathy (AAOR) is a rare entity characterized by a peripapillary gray-white ring, disruption of the outer retina within the affected area, and sudden onset of a scotoma (Figure 6). It was first described by Gass and Stern in 1995 in a 23-year-old patient who developed a sudden onset scotoma and a gray intraretinal ring corresponding to the edge of the scotoma. There was mild RAPD and no inflammatory cells visible in the anterior chamber or vitreous. The patient developed enlargement of the scotoma and narrowing of the retinal vessels within the ring for around three weeks before stabilization of the scotoma and disappearance of both the ring and the afferent pupillary defect. Development of pigmentary changes in the form of bone spicules was noted after a year within the affected area [56]. Since then, just a few cases of AAOR have been reported with variable presentation and clinical course. Fekrat et al. reported two patients treated with oral corticosteroid and subjective improvement and two cases that did not need any treatment. Two out of four eyes had an afferent pupillary defect at presentation and one patient developed scattered intraretinal bone–spicule pigment clumping after some months [57]. Luckie et al. reported a case treated with intravenous acyclovir where herpetic etiology was suspected [58]. In many of these cases, the gray-white ring around the optic nerve disappeared over time.

Many authors have speculated that AAOR is part of an acute zonal occult outer retinopathy (AZOOR) complex and is secondary to an immune reaction following a viral illness. Recently, two cases of AZOOR have been described following COVID-19 vaccination [59]. AZOOR is characterized by the sudden onset of acute scotoma with minimal ophthalmoscopic appearance and abnormal visual field and electrodiagnostic tests. AZOOR complex is characterized by overlapping clinical syndromes that are usually present in young women involving the outer retina with loss of its function. The presentation of each discrete syndrome results from an interplay of genetic, immune, and environmental triggers.

A stricter definition of AZOOR based on multimodal retinal imaging findings, including a demarcating line of progression between the involved and uninvolved retina and a trizonal pattern of chorioretinal degeneration, has been recently proposed [60].

Several AAOR and AZOOR reports describe an afferent pupillary defect and absence of inflammation in the anterior chamber or vitreous. These features have led many physicians to refer the patient to a neurologist suspecting optic nerve disease.

Shindo et al. described the case of a 30-year-old woman with recurrent episodes of sudden visual field defects and photopsia diagnosed with optic neuritis and treated with corticosteroid therapy with no improvement. MRI of the head with contrast was normal and after 15 years of mislabeling her condition, she was finally diagnosed with AZOOR [2]. Chen et al. described 20 patients, mostly females, initially diagnosed with optic neuritis or intracranial lesions who were ultimately diagnosed with AZOOR after a thorough eye examination and neuroimaging. Around 50% of them had RAPD and all of them presented with visual field defects (temporal scotoma and blind spot enlargement) and mfERG abnormalities. While the majority of these patients showed abnormal ffERG, none of these patients showed abnormalities on neuroimaging [61]. Many other AZOOR cases in the literature have been misdiagnosed as optic neuritis [1,62]. Zaslavsky et al. described the case of a 58-year-old patient with bitemporal hemianopia on visual field inspection who was diagnosed with AZOOR after evidence was presented of a normal brain MRI and areas of hyperautofluorescence in the nasal retina of each eye, corresponding to loss of the outer retina at that location [63]. On the other hand, there have been reports of AZOOR in the context of central nervous system diseases. Gass et al. published a long-term follow-up study where four out of fifty-one patients with AZOOR developed relapsing transverse myelopathy and six had multiple white matter lesions on brain MRI [64]. Hintzen et al. described a case of AZOOR and multiple sclerosis in a 30-year-old woman who developed photopsia, progressive visual field defects, hemiparesis, and hypoaesthesia. The authors suggested a common inflammatory mechanism between AZOOR and multiple sclerosis and prompted the use of electrophysiology to differentiate AZOOR from the lesions affecting the posterior visual pathway [65]. An abnormal mfERG is generally considered a diagnostic criterion for the diagnosis of AZOOR, while ffERG is less sensitive and may be normal, especially when the field defects are small. Jacobson described a case of AZOOR and cerebrospinal fluid pleocytosis, multiple brain MRI signal abnormalities, and development of symptoms compatible with acute relapsing-remitting cervical myelitis six years after visual presentation [66]. A recent case of AZOOR has been described associated with retrobulbar optic neuritis as a clinically isolated syndrome rather than clinically confirmed multiple sclerosis [61].

In conclusion, AZOOR/AAOR diagnosis is not always straightforward, and they can be mistaken for a neurologic condition thus leading to expensive and time-consuming procedures like a brain MRI. Multimodal imaging is of great help and visual field testing along with electrophysiology are essential to reach a definite diagnosis.

### 2.6. Acute Idiopathic Blind Spot Enlargement Syndrome

**Figure 7:**
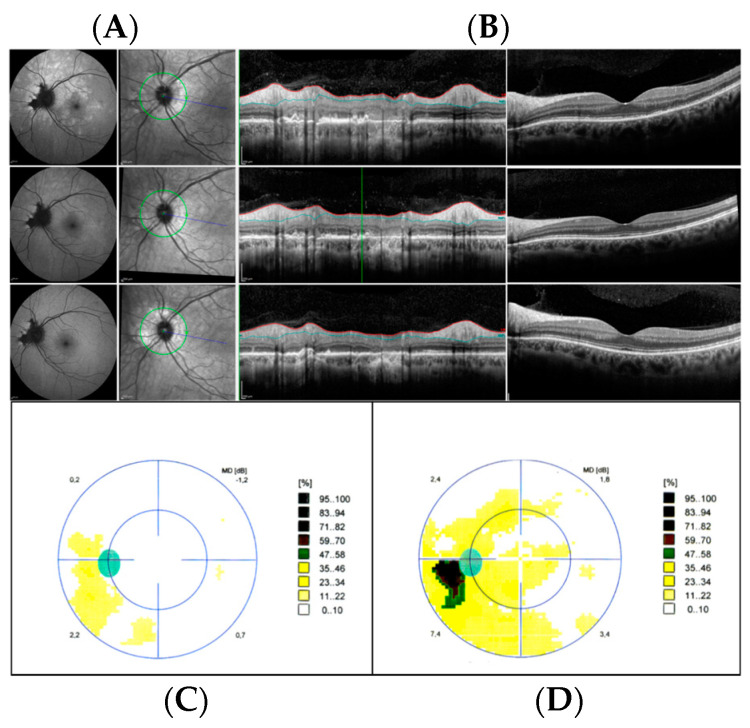
Multimodal imaging of acute idiopathic blind spot enlargement of the left eye, at presentation and during follow-up. (**A**) Baseline row shows in order: blue autofluorescence with hyperautofluorescent spots centered on the optic nerve resembling multiple evanescent white dot syndrome and peripapillary hypoautofluorescent pigmentary changes; infrared image with corresponding retinal nerve fiber layer (RNFL) optical coherence tomography (OCT) showing disruption of the outer retina and increased thickness of RNFL; macular OCT showing disruption of the outer retina. (**B**) Two-week follow-up row shows in order: faint hyperautofluorescent lesions; infrared image with corresponding RNFL OCT showing decreased thickness of RNFL and improved outer retina; macular OCT showing improvement of the outer retina. (**C**) One-month follow-up row shows in order: disappearance of the lesions on autofluorescence; almost resolved appearance of the peripapillary outer retina; resolved appearance of outer retina at the macula. (**D**) Visual field test one year before the appearance of the spots (left image) and at disease onset (right image) showing a peripapillary scotoma.

Acute idiopathic blind spot enlargement syndrome (AIBSE) is a condition affecting the peripapillary retina. It is due to dysfunction of the outer segment of the retinal photoreceptor layer usually presenting with photopsia, blurring, and dyschromatopsia in young myopic patients. The blind spot enlargement is visible on a visual field test and FAF usually shows increased autofluorescence around the optic disc (Figure 7).

AIBSE was originally described in 1988 by Hoyt et al. in seven patients with photopsia and acute enlargement of the blind spot [67]. In all the patients, a steep-edged absolute scotoma measuring 15°–20° in diameter was recorded with no evidence of disc swelling or involvement of the surrounding retina. mfERG showed a loss of peripapillary retinal function. Symptoms resolved in all patients within a year. Given the apparent absence of retinal or nerve abnormalities at fundoscopy, many of these patients were misdiagnosed as having optic neuropathy [67].

In 1984, Jampol et al. described the coincidence of enlarged blind spots with MEWDS, raising the hypothesis of blind spot enlargement being a manifestation of MEWDS [68]. Takeda et al. in the same year, and Hamed et al. in 1989, described the same association with scotoma persistence after disappearance of the dots [69,70].

In 1993, Gass hypothesized that AIBSE, MEWDS, AMN, AZOOR, and multifocal choroiditis (MFC)/punctate inner choroidopathy (PIC) were different entities of the same disease spectrum, involving the outer retina in young myopic women. Since then, many authors have used the term “AZOOR complex” or “white dot syndromes” to define this group of diseases, although distinct subcategories have been recognized [71]. Some years later, in 2000, Gass stated that the AIBSE variant with normal retinal findings was a subset of AZOOR [72].

Volpe et al. in 2001 reviewed 27 patients, all women, with acute onset of blind spot enlargement demonstrated on visual field testing. Intraocular inflammation, mild optic disc edema (inadequate to explain the blind spot enlargement), peripapillary pigmentary changes, afferent pupil defects, and white dot lesions were not uncommon. They observed that despite the resolution of these abnormalities, there was persistence of the visual field defect in all the patients. They concluded that AIBSE was a distinct entity and was characterized by symptomatic enlargement of the blind spot caused by occult peripapillary retinal dysfunction and persistence of scotoma. Despite persistent scotoma and despite six patients having recurrences, none of them showed a progressive condition [73]. As opposed to Volpe et al., Watzke and Shults in 2002 supported the idea of AIBSE being a spectrum of disease [74].

Several other small series and clinical cases have been reported in the last few years with different features, making it difficult to distinguish AIBSE from other entities, especially AZOOR [75,76,77]. A case report described the occurrence of AIBSE following influenza vaccination, similarly to our case, raising the hypothesis of immunologic etiopathogenesis [78]. Recently, a case of AIBSE was described after COVID-19 vaccination [79].

Given the variability of clinical findings, AIBSE can be misdiagnosed for other entities. When disc hyperemia or white dots are present, it can be mistaken for inflammatory diseases like MEWDS or other white dot syndromes. The development of chorioretinal lesions typical of MFC has been reported in patients with AIBSE [80]. When the retina and the disc are within normal limits on fundus biomicroscopy, it can be misdiagnosed as retrobulbar optic neuritis and referred to neuro-ophthalmologists. Indeed, in the work by Volpe et al., optic neuritis and ophthalmic migraine were common misdiagnoses [73]. Newcomb described the case of a 12-year-old patient who was followed for five months by a neuro-ophthalmologist before a definite diagnosis of AIBSE was made [81]. Moreover, the appearance of a sudden visual field defect may lead many referring physicians to ask for computed tomography or magnetic resonance imaging of the brain to rule out vascular events or neoplastic lesions.

In conclusion, AIBSE is a condition characterized by enlargement of the blind spot demonstrated on the visual field. It can present with different features and particular awareness should be raised when patients describe photopsia and enlargement of the blind spot to avoid misdiagnosis and the potential for unnecessary investigations. The discussion about considering AIBSE as a spectrum or a separate entity is still open.

### 2.7. Multiple Evanescent White Dot Syndrome

Multiple evanescent white dot syndrome (MEWDS) is an idiopathic retinal disease, first described in 1984 by Jampol et al. [68]. It typically affects young myopic females with a significant disparity between women and men (4:1). MEWDS is characterized by multiple white-yellowish dots at the level of the outer retina. Patients can experience acute onset of blurry vision, photopsia, visual field defects, and dyschromatopsia and around 30% of them have a viral prodrome some days before. This has led to the hypothesis of MEWDS being an inflammatory condition triggered by an environmental factor [82]. Recently, many MEWDS cases have been reported in association with COVID-19 infection and COVID-19 vaccination [83,84]. It is usually self-resolving in around 6–12 weeks with complete restoration of the retina structure and disappearance of the symptoms. However, some patients may experience slower visual recovery, especially if they are young or present with worse visual acuity [85].

**Figure 8:**
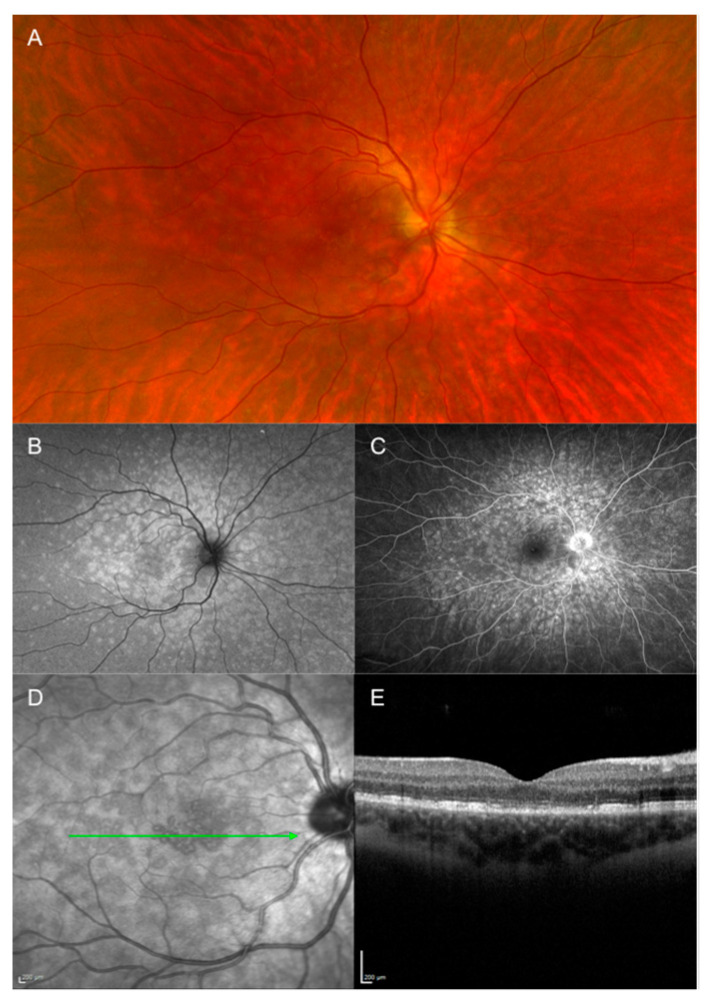
Multimodal imaging of multiple evanescent white dot syndrome of the right eye at presentation. (**A**) Pseudocolor fundus photography shows some faint yellowish lesions at the posterior pole. (**B**) Green autofluorescence shows hyperautofluorescent spots spread beyond the vascular arcades. (**C**) Fluorescein angiography shows hyperfluorescent lesions in a wreath-like pattern and hot disc. (**D**) Near-infrared image shows the same lesions with enhanced granularity at the fovea. (**E**) Structural optical coherence tomography scan corresponding to the green line in panel (**D**) showing outer retina disruption.

MEWDS is a typically unilateral condition, although bilateral and recurrent cases have been described [86]. Fundus examination is characterized by white-yellowish spots and dots at the posterior pole and around the optic nerve. The other pathognomonic sign is foveal granularity. The spots can be very faint if some days have passed and can even be lacking entirely [87]. OCT shows that the spots colocalize with focal disruption of the outer retina, in the RPE-photoreceptor complex, whereas the dots are extensions of the ellipsoid zone towards the inner retina. Fundus autofluorescence is of paramount importance to visualize both dots and spots, which are shown as hyperautofluorescent lesions. NIR imaging discloses them as roundish grayish lesions. On FA, spots and dots are characterized by a typical wreath-like configuration and hyperfluorescence from early to late phases of the dye test (Figure 8). On ICGA, they are characterized by hypofluorescence in mid and late phases [86].

On the basis of multimodal imaging, many hypotheses on the origin of these lesions have been proposed. It has not been fully elucidated if MEWDS is a primary photoreceptoritis or a RPE disease that secondarily involves the photoreceptors. Some authors supported the hypothesis of MEWDS being a disease of the choriocapillaris [82]. However, recently, optical coherence tomography angiography has shown no signs of disease on the choriocapillaris in MEWDS [88]. No specific baseline multimodal imaging features have been associated with visual outcomes or the clinical course of MEWDS [85].

Visual field testing can show different patterns of defects, with the two most common being an enlarged blind spot and a centrocecal scotoma. A few studies have been conducted on MEWDS and electroretinography. Oh et al. found that in the acute phase, all of their patients had focal areas of reduced retinal function on the mfERG first order responses corresponding to enlargement of the blind spot. Four patients also demonstrated diffuse loss of b-waves with greater loss around the blind spot. In the recovery phase, all eyes with MEWDS demonstrated complete recovery of retinal function on mfERG first order responses [89]. In another work by Horiguchi et al., they describe two patients with different clinical presentations. The one with central scotoma had a more reduced amplitude in mfERG than in ffERG, and its recovery was much slower in the former. In the patient with blind spot enlargement, the amplitude of mfERG was more reduced in the peripapillary region than in the macula, and the recovery of the amplitude slower around the disc [90]. The differential diagnosis of MEWDS should be made with the other entities in the white dot syndrome spectrum and with neuroophthalmological disorders. Acute idiopathic blind spot enlargement is characterized by enlargement of the blind spot and sometimes by evidence of hyperautofluorescent spots. It is considered as a subtype of MEWDS [91], although some authors recognize them as different diseases [77]. The blind spot enlargement in MEWDS resolves over time, while in AIBSE resolution of the visual field defect does not always occur. Other inflammatory conditions like acute multifocal posterior placoid epitheliopathy, acute retinal pigment epitheliopathy, or birdshot chorioretinopathy can be initially mistaken for MEWDS but multimodal imaging helps in distinguishing them. Recent studies differentiate an idiopathic or primary form of MEWDS from a secondary form that is seen in association with other conditions, not necessarily uveitic, affecting the posterior segment of the eye, like MFC/PIC, high myopia, or previous vitreoretinal surgery for rhegmatogenous retinal detachment [92,93,94]. A recent work by Russel et al. described disorders characterized by widespread grayish-white spots associated with hyperautofluorescence on FA and disruption of the ellipsoid zone on OCT, masquerading as MEWDS. These disorders included syphilis, lymphoma, MFC, idiopathic retinal phlebitis, AZOOR, sarcoidosis, tuberculosis, and cancer-associated retinopathy [94].

Among the neuro-ophthalmological conditions mimicking MEWDS, optic neuritis had the same presenting symptoms of unexplained unilateral visual loss in a young woman. RAPD and dyschromatopsia can be present in both MEWDS and optic neuritis, respectively, due to retinal or optic nerve dysfunction. However, a history of viral prodrome, lack of pain at ocular movement, and multimodal imaging are of great help in the diagnosis. Dodwell et al. described five patients with MEWDS where the subtlety of the white spots made the diagnosis difficult. They had variable presentations of primary nerve disease with RAPD, optic disc edema, and dyschromatopsia. Two patients were first diagnosed with “optic neuritis”, two with “retrobulbar optic neuritis”, and one with “optic nerve edema and vitreitis” [95]. Khaleeli et al. described a case of MEWDS with paracentral red desaturation and patchy scotoma on confrontation that was referred to a neurologist with suspected neuritis. However, multimodal imaging again helped to reach the correct diagnosis [96]. Pellegrini and Interlandi described a patient with MEWDS misdiagnosed as optic neuritis who underwent MRI, which ultimately resulted negative. The authors highlighted the importance of distinguishing these two entities to avoid unnecessary and expensive radiologic testing [3]. To note, a case of MEWDS associated with multiple sclerosis has been described by a single report, where the authors hypothesized a common inflammatory mechanism between MEWDS and multiple sclerosis, despite admitting that the association could have represented a coincidence [97].

## 3. Discussion

Acute-onset retinal conditions may present with subtle or normal-appearing fundus examination and thus are referred to neurologists as possible neurological conditions such as AON. Timely recognition of these cases is therefore important for optimal management [96]. These retinal conditions mostly affect young adults and have common presenting symptoms and similar clinical courses. However, they have distinct and peculiar imaging patterns that are of help in the differential diagnosis.

Multimodal retinal imaging including OCT, NIR, FAF, color fundus photography, and possibly retinal angiography play a crucial role in the diagnosis of these retinal diseases. These specific investigations are rarely available in a neurology clinic or in a general ophthalmologist’s office, but clinicians should be familiar with their interpretation and know when and what to request.

While a detailed differential diagnosis between these retinal conditions and other acquired sudden-onset retinal conditions is not the aim of the present manuscript, infectious conditions such as syphilis and either latent or active tuberculosis presenting with placoid lesions on the retina should be kept in mind [17].

In cases of unilateral or bilateral unexplained visual loss, retinal disease should be included in the differential diagnosis of neurological vision loss, as either an optic neuropathy or as a “central” disorder. When the visual loss is monocular, the differential diagnosis between retinal disorder and optic neuropathy is usually straightforward when obvious retinal abnormalities are found. In the case of binocular vision loss and normal-appearing fundus examination, neurological causes can often be identified by visual field defect patterns. By contrast, a lack of optic nerve enhancement in presumed AON on orbital and brain post-contrast MRI may be suggestive of a retinal condition.

Clinicians should suspect acute retinal disease mimicking AON in patients with acute painless visual loss. The presence of ophthalmoscopically visible lesions in the fundus should of course prompt multimodal retinal imaging. The absence of visible pathology in the fundus, however, should not refrain the clinician from considering multimodal retinal imaging, especially when symptoms (e.g., phosphenes or photopsias, metamorphopsia, ring or central scotoma, blind spot enlargement without papilledema), signs (lack of RAPD), or neuroimaging (lack of optic nerve enhancement or demyelinating white matter lesions for multiple sclerosis) suggest a diagnosis other than AON.

## 4. Conclusions

Misdiagnosis of acute retinal disorder as AON can lead to inappropriate and potentially expensive and unnecessary neurologic evaluations. A detailed review of signs and symptoms along with multimodal retinal imaging are essential in detecting these retinal conditions and in their differential diagnosis.

## Data Availability

Not applicable.

## Abbreviations

AAOR	acute annular outer retinopathy
AIBSE	acute idiopathic blind spot enlargement
AIM	acute idiopathic maculopathy
AMN	acute macular neuroretinopathy
AON	acute optic neuritis
ARPE	acute retinal pigment epitheliitis
AZOOR	acute zonal occult outer retinopathy
DCP	deep capillary plexus
ELM	external limiting membrane
ERG	electroretinogram
EZ/IZ	ellipsoid zone/interdigitation zone
FA	fluorescein angiography
FAF	fundus autofluorescence
ICGA	indocyanine green angiography
INL	inner nuclear layer
IPL	inner plexiform layer
MEWDS	multiple evanescent white dot syndrome
MFC/PIC	multifocal choroiditis/punctate inner choroidopathy
MRI	magnetic resonance imaging
NIR	near-infrared
OCT	optical coherence tomography
ONL	outer nuclear layer
OPL	outer plexiform layer
PAMM	paracentral acute middle maculopathy
RAPD	relative afferent pupillary defect
RPE	retinal pigment epithelium
